# Behavioral Intruder Detection Based on Browsing Patterns with Automated Grouping of Requested Webpages

**DOI:** 10.3390/s26020473

**Published:** 2026-01-11

**Authors:** Artur Wilczek, Konrad Ciecierski, Mariusz Kamola

**Affiliations:** R&D Division, NASK National Research Institute, 01-045 Warszawa, Poland; artur.wilczek@nask.pl (A.W.); konrad.ciecierski@nask.pl (K.C.)

**Keywords:** Siamese network, intruder detection, weblog, low-rank factorization

## Abstract

Impersonation attacks causing online fraud are a growing challenge for digital services, demanding the integration of biometric and behavioral factors into traditional authentication methods. Behavioral impersonation detection during online sessions is particularly critical for online banking, and the existing solutions focus mostly on mouse and keyboard dynamics. We study behavioral patterns extracted from standard web-server logs and claim that our methods are applicable in a banking scenario. Using a Siamese neural network, we classify pairs of web sessions from the same user with 90% accuracy. Experiments conducted on real-world intranet weblogs, serving as a proxy for banking data, highlight challenges in filtering and aggregating data. To address variability in website technologies and browsing behaviors, we introduce an automated procedure for grouping requested pages based on a low-rank approximation of the user browsing matrix. This approach consistently improves classification accuracy while reducing reliance on costly, error-prone manual log analysis, offering a scalable, viable approach for fraud detection in online services.

## 1. Introduction

Impersonation fraud in online banking is just one example, though an extreme one, of an impostor scam that results in the interception of a customer’s credentials. The problem grows yearly as new communication media and new cheating scenarios appear, as reported by [1,2]. The impostor scam has been identified in [3] (2023) as one of its four top challenges, ranking far ahead of other fraud types and resulting in USD 2.6 bln in direct losses in 2022. Wary of the threat, banks and national security agencies roll out appropriate fraud-detection software and run customer campaigns. However, almost every new money-related and public activity, be it a refund scheme, charity fundraising, or tax exemption law, can become an original vector of attack. Therefore, the ultimate line of defense lies in the banking application backend, where various systems scrutinize customers for actions that do not seem their own.

Consequently, online services, banks in particular, are rolling out extra user authentication and emulation detection methods, like one-time passwords and human-friendly simple tasks—cf. [4,5]. However, behavioral authenticity verification is on the rise because it relieves users from extra authentication effort and reveals their individual traits for automated identity verification.

Behavioral detection of an intruder that has falsely logged into a web application may use modalities such as mouse movements, keyboard typing, sequence of pages visited, etc.—individually or combined. Data collection of fine-grained behavior, such as mouse or keyboard, is performed on the client device, thus introducing a considerable overhead, both in client application code loading and execution phases. Last but not least, it raises privacy concerns because user behavioral features needed by, e.g., online banking intrusion detection systems, once collected, may later be used to identify the same users in other or third-party services where they want to stay anonymous.

In this study, we design and verify an algorithm that may be used to detect an impostor interacting with a website and trying to carry out an impersonation attack. We achieve this through an examination of web-server activity logs only. The core model used by the algorithm is a Siamese neural network because it can handle training scenarios with few samples and because such a model does not perform aggregation of user behavior into a feature vector of any sort. Instead, the model learns transition patterns between visited webpages that distinguish users the most. Such a model can be applied only to compare the current web session to previous ones. Therefore, it has limited applications beyond the website for which it was trained. Siamese networks were initially used for comparing written signatures by [6] and have since become a recognized modeling approach.

The abilities of the proposed model depend strongly on the way a website is built. In particular, the architecture and framework chosen for the client side determine the amount and value of data in the log. Also, the inherent responsiveness of the framework used on a website affects model operating conditions. However, when used in an ensemble with other approaches, the model can provide useful hints about user behavior. Finally, to circumvent the need for expert knowledge in preliminary log structure analysis, reformatting, and filtering, we propose an automated approach for grouping webpages based on low-rank matrix factorization.

The rest of the paper is divided into three sections. In Section 2, we review similar work, and in Section 3, we present our data, the Siamese model, and an automated webpage request grouping proposal. Section 4 contains the main results and their discussion. We conclude our work in Section 5.

## 2. Related Work

The aim of the work presented here falls in a vast category of user identification tasks performed in specific scenarios, such as user authentication, additional verification of an already authenticated user, or matching a user’s multiple identities. User authentication implies comparing the presented credentials against a database, which must at least partially be static to serve as multifactor authentication in the first stage. Additional user verification, also termed continuous authentication, comprises surveillance of an individual who has logged in, in search of suspicious behavior; i.e., that differs from his/her previously observed traits. This set of historical activity, called the gallery [7], may contain raw samples of former interactions or higher-level user features. Finally, matching user profiles aims to detect duplicate identities created by the same user and associate them based on similar behavior.

Accomplishing the above goals requires adequate types of data as well as user activity modeling; a comprehensive overview of the subject can be found e.g., in these works [8,9,10]. The traits used for verification are broadly divided by [9] into physiological and behavioral ones. Features of the face, ear, iris, fingerprints, and other body parts, as well as elementary and body signals (EEG, ECG), are considered physiological traits, while body part and equipment movement (gait, keystroke, speaking etc.) are considered behavior-related ones. Collecting physiological traits requires specialized hardware, which is why it is addressed so sparingly in papers. Most of the studies feature mouse and keyboard behavioral traits, as the works reported below that have inspired us in our research.

### 2.1. User Input-Driven Approaches

Mouse dynamics data lie closest to handwriting biometrics in terms of frequency and semantic level—yet their acquisition does not require specialized hardware or software privileges. That is why it is widely used as a standalone method or as a modality for user identification. Yi et al. [11] show, in an experiment, that mouse dynamics are highly user-specific and clearly depend on the user’s emotional state, and that these two aspects can be processed separately and for different purposes, in particular for user impersonation detection. The authors use a random forest model with cursor velocities as its input. Wang et al. [12] also use random forests to build individual user profiles, but also include specific, non-semantic web session attributes, such as typical activity hours, the fraction of a webpage read, or user display ratio. Both studies were conducted with volunteer users.

Keyboard dynamics technically provide samples at a lower frequency than a mouse, but the information per sample is far richer due to the number of keys and differing physical and logical key layouts. Similarly to mouse data studies, the published works are mainly carried out as controlled experiments where a number of users are prompted to type out passphrases that are their own, others’, or random ones. Giot et al. [13] record intervals between key events for each letter of a fixed password and show that typing manner distinguishes users in a control group. The Siamese network (described in detail later) is the chosen model that compares two samples and returns the likelihood that they were typed by the same person. This approach has been extended by [14] by making the controlled group type masked passwords and inferring user identity from the way selected password characters are typed in. The model class used is the Siamese network again. Recently, models with convolutional and recurrent neural layers topped the list of the 2023 Keystroke Verification Challenge contest, as summarized by [15]. Interestingly, an approach that is the opposite of applying deep neural networks with memory layers is presented by [16]. Named ITAD, for instance-based tail area density, it operates by comparing timings of keyboard events in the current session vs. cumulative distribution functions (CDF) formed by its counterparts in the user’s gallery i.e., the history of past interactions. Evidence exists that ITAD can easily deliver business-grade performance, yet with almost no preliminary data processing, such as model training.

Varnosfaderani et al. [17] jointly analyze mouse and keyboard behavior to identify users who have registered with multiple identities. In order to discover them, the authors apply a variational autoencoder network, i.e., a model that is required to replicate its input on its output, but an information bottleneck is designed in the normally distributed middle layer so that effective compression and behavior abstraction must happen there if the model is to reach the goal. Statistics of mouse clicks and keyboard hits are presented at model input, along with some browsing-specific data, such as the last login time, the number of backspaces during login, and the browser type. The experiment was carried out in a controlled environment of volunteer users of GWDR, a German academic services aggregator and single authentication point. Consequently, a sequence of services visited after login was added to the model input. The user-generated data was augmented to reach 30,000 samples; the model used did not contain recurrent layers. It appears that those modalities get merged directly at the model input, which is rather an unconventional approach. According to [9], the respective model parts usually process separate modalities. Their fusion occurs either at the level of feature vectors (i.e., somewhere in the middle of the deep neural network architecture) or only at score level (i.e., at the very last phase judging user identity).

### 2.2. User Navigation-Driven Approaches

The study by [17] introduces the topic of user identification based solely on activity in web-server logs. It receives well-deserved attention, probably because this user activity data is abundant, semantically rich, and available instantly. Unlike, e.g., keyboard or mouse dynamics, collecting it does not require the development and injection of JavaScript code, which often raises privacy, security, and performance concerns—concerns expressed by both the service provider and its customers. Introducing continuous user authentication by weblog analysis is non-intrusive. It can be applied as the first step in service improvement, with a low entry barrier, or added later as reinforcement to an existing user-scoring system. However standard the weblog format is, the actual level of detail depends strongly on the page design—but the minimum information available comprises a timestamp, the browser type, the HTTP request type, and the URL of the resource a user interacts with. The level of semantic information extracted from those data, especially URLs, varies across approaches in the literature. In one of the early works, ref. [18] distinguishes several classes of user actions, namely, interactions with specific website functionality. Sequences of actions from Markov models, extracted for individual users, are used for continuous identification. However, such a modeling approach is feasible only for small classes and short browsing history. In later works, ref. [19] likewise reduce user activity space to typical actions (popup, view, search, login, submit etc.) but enrich model input by percent of page viewed, timestamps, or display ratio, similarly to [12]. The model class used was XGBoost, trained on 18 selected users’ natural activity over a year. Jin et al. [20] employ SimHash, a concept of efficient data hashing, cf. [21], to histograms of visited webpages’ features to create unique user profiles. The features considered are time spent on a page, user device type, webpage address, webpage rank in the network, and so on. The study by Sudhakar et al. [22] is similarly based on weblog analysis alone, but the authors point out other relevant traits: hovering the mouse over a link, text selection, window scrolling speed, bookmarking, saving, and printing a page. They use long short-term memory (LSTM) layer at the model input, achieving promising results on an example public dataset.

Another approach to user identification based on weblog analysis, by [23], also uses the LSTM layer but stands out because, first, it has been successfully applied to broad real-world data from many users and with versatile websites, and second, because it is based only on high-level semantics of URLs in sequence. The semantics were retrieved by URL address tokenization and representation as a bag of embeddings. The LSTM layer forms a part of a Siamese network. Since the task was to match duplicate user identities based on their behavior, the authors report a notorious imbalance in the positive/negative example counts when pairs of samples for model input are drawn at random. It is relatively unlikely that a random pair will belong to the same individual—which the authors address by proper weighing applied to training loss and performance evaluation functions.

A dual approach to user identification by [24] is based on fingerprinting webpages rather than users. Webpage features get attributed by sequence patterns, i.e., explicit rules that capture page-visiting patterns by all users. The rules are expressed in temporal logic and have been tested by the authors on logs of users browsing commercial product review pages. Finally, users are distinguished on the fly by the fingerprints of the pages they are currently browsing, without any other personal info being stored. The authors’ final best classification model is random forest.

## 3. Materials and Methods

### 3.1. The Dataset and Pre-Processing

The research reported here was carried out on a dataset comprising WWW server logs from an intranet site within an organization. We have limited the scope of our study to just one modality for both practical and strategic reasons: first, analysis of such rich and self-contained data is prospective and popular, as was shown above; second, our research was of complementary character to developed commercial models using other modalities. Yet we claim that an intranet site can serve as a good proxy for a real online banking service: users must log in to perform authorized operations, such as downloading specific documents or posting data via HTML forms. Their interaction with services in both scenarios falls in a range of social behavior—from almost none while logging in, to fully interpersonal while sending a message, or money. Also, the degree of similarity between the two scenarios varies accordingly. Therefore, the model development procedure for an online banking website should derive from our approach with varying degree of confidence. Functional and technical similarities between the intranet website and selected banking websites have been provided in Table 1. The considered banks have 4 to 11 million customers, most of them using their e-banking services and competing in the same market. The table shows a bigger picture that online banks use a plethora of implementation approaches to provide typical functionalities. Many of these technical solutions work similarly in terms of user interface and network communication to the operation of Confluence, the framework that was used to run the corporate website of our interest. Two common observations are of particular interest and consequence to further modeling. First, user activity can be sent to the server after he/she has completed a form—but also in smaller chunks, during typing. The result is that a weblog can contain activity records of varying granularity. Second, the communication is mostly directed to one backend service, meaning that most activity data is stored in one place. (Otherwise, preliminary aggregation of logs from accompanying services has to be done before feeding them into the model.)

The user activities subject to our analysis spanned from February to June 2023, when the organization and the surrounding social and business conditions were stable. Let us define a single weblog record as a tuple $v_{i}=\left(c,t,U\right)$, where *c* is a user identifier, *t* is a timestamp, and *U* is a URL string. Records are ordered chronologically.

**Figure 1:**
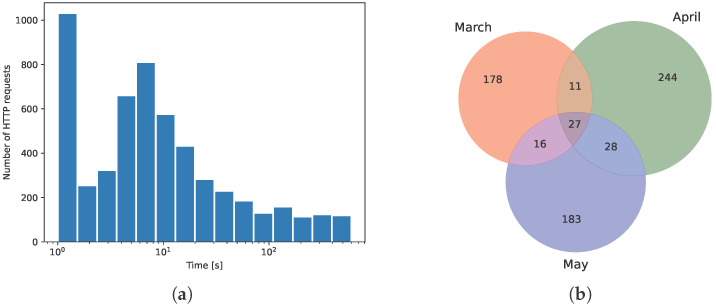
(**a**) Distribution of HTTP request inter-arrival time and (**b**) set intersection of unique URLs requested in three subsequent months, with their sizes.

Figure 1 demonstrates two main phenomena in the data that obstruct behavioral modeling: machine-to-machine (m2m) communication and user interest drift. The earlier one is caused by JavaScript (JS) code being downloaded by a browser from a webpage and executed locally. The pattern is now commonplace; the JS can trigger extra HTTP requests in response to more subtle user behavior, such as hovering the mouse cursor over a hyperlink, or page scrolling, thus providing extra behavioral data directly to the weblog; cf. [22]. But on the other hand, m2m can litter the weblog with records unrelated to the user’s spontaneous actions, and so obstruct analysis. For instance, in Figure 1, one can observe an anomaly in the distribution, just above the 0100 sec. bar, which may be generated by automated keepalive requests. Similarly, automated requests but with different timing have also been spotted on the Bank#2 (cf. Table 1) website. Due to anomalies like that, the distribution in Figure 1a does not match any class of probability distributions typically used to model stochastic arrival processes (Poisson, Weibull, etc.) frequently used in telecommunications traffic analysis.

The phenomenon of user interest drift is shown in Figure 1b; the circles’ intersecting areas represent the number of common unique URLs in the subsequent three months. The non-stationary aspect of user activity is evident and persistent. It may be driven by many factors: new content placed on the website, users’ coordinated activity caused by company management directives, and changes in the technology behind some webpages on the site. Although real critical applications, like online banking, do not exhibit such a degree of content variability as in our case, it is reported that their front-end components have a lifecycle of only 18 months and get replaced individually [25]. This results in considerably abrupt dynamics in banking website content and operation, which should be handled by re-training the model.

We filtered out m2m communication in the preparatory stage by setting the maximum accepted inter-arrival time to 256 s. By doing so, rare keepalive requests that occupy the distribution tail (not shown in Figure 1a; observe semilog axes scale) were removed. The remaining records (which originally did not contain a session ID) can now be neatly arranged into sessions of reasonable duration. The timeout value was intentionally aligned to 256. Such a value is, on one hand, similar to a typical 5 min online banking session expiry time and, on the other hand, being a power of two, it matches the intended logarithmic dynamics encoding in our model.

We address the interest drift as shown in Figure 1b using two alternative approaches, both resulting in a representation of the URL string *U* as a vector x of constant size. The first and original one is to group URLs into semantic categories—and from that time on, to see user activity in terms of categories visited rather than individual URLs. The specific procedure in our case was as follows.

1. Expert inspection and cleaning of weblogs. Entries that carry noise or information excessive to our interest—like request tokens, sequence identifiers, and similar machine-generated artifacts—are identified by manual log browsing and filtered out by regular expression matching.
2. URL distribution tail inspection. Rare URLs are examined manually with the aim of finding unusual human requests or remaining machine-generated activity that has been overlooked previously. Suppressing URL distribution tail reduces web requests versatility, eliminating exotic request classes that would impede further machine learning process.
3. Assessment of the number of possible request classes vs. the number of samples in each class and for each user. Requests are assigned intents by an expert; an intent is finally encoded by a set of regular expressions, usually matching a part of the URL. It is an iterative procedure, aiming to end up with a manageable number of meaningful classes and with enough samples in each class.

In the case of our dataset, we eventually distinguished eleven broad categories of user activity, ordered below from more specific to more general:

- log in;
- log out;
- liking someone’s post;
- displaying a PDF document;
- displaying a DOCX document;
- browsing employees’ profiles;
- searching employees;
- free text search on website;
- browsing yet unloaded webpage part;
- browsing yet unloaded tab on a webpage;
- browsing any other webpage.

Quite a few of the above activities can be considered functional counterparts of online banking actions; cf. Table 1. The remaining ones cover other user actions in broader categories.

Performing such semi-manual data curation is laborious and requires a trained expert. It also provides considerable insight into requests, both qualitative and quantitative. While informative, the approach may, in certain circumstances, reveal too much about what is happening in the network and should be applied with caution, regardless of its effectiveness.

The second analytic approach is to automatically organize requested webpages into groups that represent frequent browsing patterns. It is presented in detail in Section 3.3.

### 3.2. Siamese Network

The term “Siamese network” was coined in [6], but the idea of employing twin neural networks for time-series analysis dates back to the late 1980s in [26]. The essence of the idea is to train a network to distinguish between pairs of input samples that belong to the same or different categories. Formally, a network performs a mapping y=F(x) of input vector x into output vector y. The network always operates on pairs of input vectors $\left(x,x^{\prime }\right)$—hence its name. In the training procedure, a mapping is sought that minimizes the distance $d(y,y^{\prime })$ of its output vectors for x and $x^{\prime }$ being in the same class, and maximizes the distance otherwise. The function d(·) can be a classic one, e.g., Euclidean or cosine, or calculated by a non-linear formula, for instance, by another neural network layer. Thus, instead of an unrealistic one-class-per-user classification task, the Siamese network provides simple similarity metrics for pairs of user-generated inputs. The approach turns out to be useful, especially when the number of samples per user is small (as in our case)—yet the number of pairs of samples is large enough to make proper machine learning viable.

**Figure 2:**
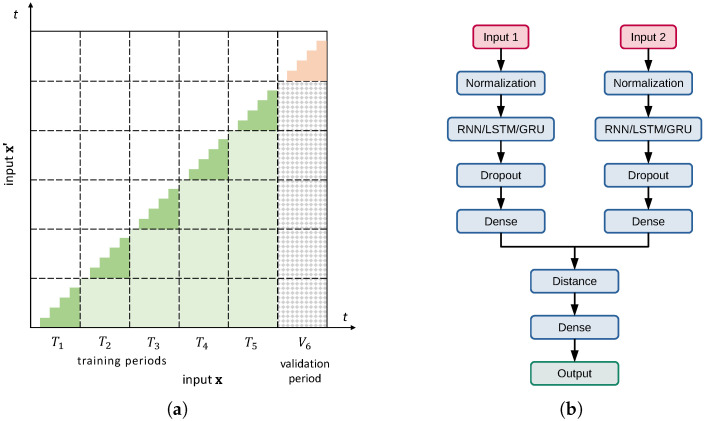
(**a**) Partitioning of sessions into training (green) and validation (pink) pairs — with light green and chequered areas as training and validation variants; (**b**) the architecture of model used.

Specifically, in order to detect temporal patterns in user activities, we follow [22,23] and provide sessions. A session is encoded as a sequence of vectorized identifiers of subsequent pages visited by a user, $x=(x_{1},x_{2},\ldots )$, and $x^{\prime }$ likewise. Such a pair of same-length sequences of uniform vectors constitutes a single sample. To handle such sequences, a layer with memory (RNN/LSTM/GRU) is placed immediately after the input normalization layer. The last two layers are the dropout and densely connected ReLU; cf. Figure 2b. It is, in fact, a minimal, reasonable configuration that can process sequences, transform them into vector output, and include an elementary protection mechanism against overfitting. Finally, distance d(·), i.e., the model output, gets calculated by the last dense layer containing, in fact, a single perceptron with a sigmoid activation function. The perceptron input z is a vector of pairwise distances between the outputs of two branches, $z_{i}=\left|y_{i}-y_{i}^{\prime }\right|$.

Unlike [23], we vectorize page identifiers by one-hot encoding visited URL groups rather than using word2vec URL embeddings—because we do not find them well suited to our weblogs, which may contain many page IDs generated automatically by a content management system (CMS). As it turns out, such a circumstance is quite common and distinguishes our case from the wide web address analysis performed in [23], where meaningful URLs prevail. For similar reasons, we employ neither user agent data nor page rank as extra input features, as in [24]; both mentioned studies aim at identifying user sessions in a wide space of URLs, which calls for semantic insight into addresses for the sake of their diversity, and the processing of user device features, for the sake of user diversity. As regards URL semantics, we considered it superfluous and uninformative in the case of our particular intranet service; as regards client device metadata, first, the devices were only corporate personal computers, and second, collecting such data would require website server reconfiguration that was administratively prohibited.

For the purpose of network training, we partitioned users’ sessions chronologically into six periods, or folds, of approximately equal cardinality. During model preparation, we used one period for validation and the remaining periods for proper training. An exemplary partition is shown in Figure 2a. There, the last period is used for validation, which corresponds to a hypothetical deployment scenario in which the model is first trained on past user sessions and then used for inference on new vs. old data. However, technically, data chronology should not matter because the user request process is not self-dependent, and any drift comes from external factors, which are considered here as unobservable disturbances. Their nature is, to some extent, revealed in Figure 1b. The unique and common URLs in subsequent months exhibit a clear, symmetrical pattern, with no particular preference for URL variety in the last month, May. For this reason, we also consider partitioning with other periods used for validation in our experiments.

Each training or validation sample is, in fact, a pair of sessions, $\left(x,x^{\prime }\right)$. The pairs can be picked up from the available dataset using various approaches. Our base scenario is to sample unique pairs of sessions within a given period, i.e., $x\in T_{i}$ and $x^{\prime }\in T_{i}$, and $x\geq x^{\prime }$ (with chronological order assumed). Training subsets extracted this way are depicted in Figure 2a as a series of ragged green triangles under the diagonal. Such a practice of training by using different users’ samples that are neighbors in some sense (here, chronologically) has already been proven effective in Siamese model training by [27,28], by presenting “closest-negative” pairs on model input. This approach is not only more challenging in terms of model working; it also severely limits the number of available training pairs. Note in Figure 2a that the light green large area covers potential training pairs if one is going to drop the rigor of chronological bucketing. On the other hand, the bucketing approach may serve better if there is trend in the data—which is exactly our case, cf. Figure 1b.

It is also worth mentioning another training case where the overall number of sessions is really low, so that following strictly such partitioning may yield a number of unique samples, i.e., session pairs, still inadequate for proper training of more complex models. Getting extra data from other services is an option only if they have exactly the same architecture, but the final obstacle is user privacy protection laws. On the other hand, we see no reasonable in-house data augmentation policy here. However, in such a situation, we recommend also utilizing the mixed pairs drawn from training and validation data (chequered area in Figure 2b) for model validation. They conform with the deployment scenario as well, because a session from the remote past (i.e., used for training) may serve as well as a specimen of user behavior as a recent sample. We claim that the recycling of old sessions in this manner, while formally being outside the model training paradigm not to use training data for evaluation, is viable in practice. This is because both training and evaluation phases are carried out on pairs, and we guarantee that in the validation phase, at least one session in a pair is brand new, which means that the pair constitutes a new stimulus in model evaluation effectively. To our knowledge, no studies exist that evaluate the potential impact of such an approach on time-series data in a quantitative way, which also implies that it should be applied with care.

Following the recent study by [29], one observes that both the number of users and the number of samples per user are important for successful Siamese network training. However, a bigger number of users helps the model generalize the differences between users, therefore boosting its sensitivity to detect an impostor, which is, in our case, more important than model specificity. By applying the criteria that a user must have logged in at least 24 times, and the session must contain at least five page requests, we selected 10 users as suitable to provide model training data.

### 3.3. Grouping Automation

As shown, machine-to-machine communication, user interest drift, and website updates or upgrades all contribute to the non-stationary nature of individual user behavior. Many of those factors can be effectively mitigated by expert insight into weblogs, resulting in requested webpages being a priori clustered into groups. However, it requires adequate domain knowledge, continuous supervision, and data curation, which may turn out inconvenient, ineffective, error-prone and costly, rendering our approach not viable as a ready-to-use product, thus jeopardizing its massive adoption for intrusion detection tasks.

**Figure 3:**
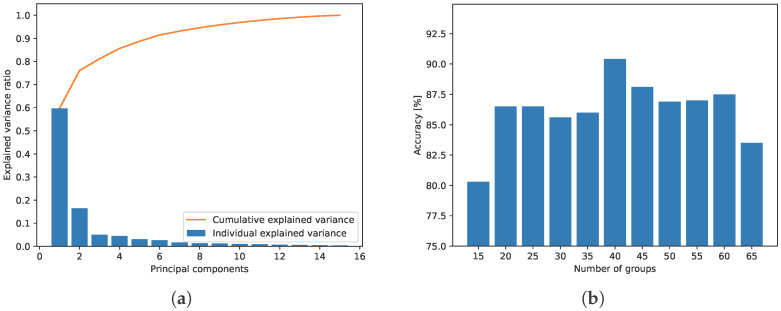
(**a**) Explained variance ratio for subsequent principal components; (**b**) classification performance with regard to number of groups *k*.

As a solution to that, we propose the approach of automatic URL grouping by low-rank factorization of the user’s browsing history, a technique widely used, e.g., in recommendation systems by [30]. Let $r_{ij}$ be the number of URL $U_{i}$ visits by user $c_{j}$, forming a so-called ranking matrix $R_{M\times N}$. *M* and *N* denote the number of unique URLs and the number of users, respectively, and the matrix itself collects user preferences to visit URLs. Here we assume that R, i.e., browsing statistics deprived of chronology, contains patterns common with regard to users and/or URLs. Therefore, R can be compressed, and the problem involves finding matrices $F_{M\times K}$ and $P_{K\times N}$ such that R≈FP and *K* is less (preferably, much less) than *M* and *N*. Matrices F and P are said to contain URL features and users’ preferences, respectively, in a space of *K* dimensions. By adequate choice of *K*, we can strike the right balance between fidelity to users’ actions and generalization capability. Usually, the choice of *K* is based on the explained variance ratio of the original data R with regard to *K*, as shown in Figure 3a. Commonly, the reduced dimension is set so that about 90% of the original variance is explained—which results in K=6 in the case of our activity dataset.

**Figure 4:**
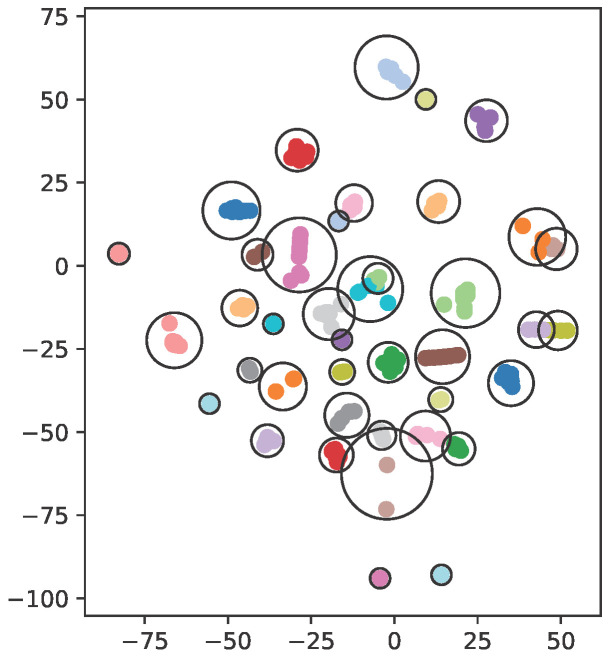
t-SNE projection of *k*-means URL clustering into 40 groups.

Once URL attributes get projected onto that *K*-dimensional feature space, we cluster them into an arbitrary number of *k* groups using the *k*-means grouping algorithm. An exemplary clustering of URLs into 40 groups is presented in Figure 4, after t-SNE mapping into a two-dimensional plane. Each group has been marked with a different color and encircled for presentation clarity. URLs in the same group have similar features, although abstract ones, and match the preferences of certain users who have visited them. Unlike in manual grouping, we cannot securely assign a physical interpretation to the features. However, if we look closely at the formed groups, we can easily see the certain patterns they follow. Some of the most populous ones contain requests that were made just once or a few times. The semantics of these requests are often not related to each other in any way. For instance, a group of requests for news on the website has emerged—such content is very coherent, but because news gets added every now and then, it is not the type of content that users return to. Another group of requests of a similar nature contains requests for viewing or downloading PDF files. As mentioned earlier, these files are not directly related to each other but denote specific, distinct user actions. Other examples of groups contain, e.g., common actions, such as a sequence in the login process, or all requests related to a particular project run by the organization. Such groups, however, are marginal.

The original dataset with actual URLs replaced by IDs of the 40 groups, and after further anonymization steps, is made available to the public, along with Python 3.9 code for model creation and training. The reference is provided in the “Data Availability Statement” paragraph.

## 4. Results and Discussion

After extensive trials with various neural network model structures and metaparameters, in the following sections, we compare and discuss in detail the most relevant and informative results. We consider the method of grouping one of our main research contributions, while the model metaparameters have undergone a typical check. Specifically, all experiments use a uniform model structure, with a Siamese network branch consisting of an input normalization layer, a memory layer (RNN, LSTM or GRU) with 32 units, a dropout layer with a rate value of 0.3, and a dense layer with 64 units and the ReLU activation function. As for the training parameters, in our case, the learning rate was set to 0.0001 (with the Adam optimizer), the batch size was set to 256, and the number of epochs was set to 30.

The discussion below follows the way the model data have been prepared.

### 4.1. Models Trained on Data Grouped Manually with Expert Insight

A compilation of the performance of various tested models is provided in Table 2. Model accuracy, i.e., the fraction of correctly identified validation samples, is the performance measure of our choice because it does not favor any output class. However, it matters which data subset is used for validation. Our models get evaluated by “k-fold” validation, i.e., cross-validation with validation data taken from one of the six equal subsets, ordered by time. Consequently, results in column 6 refer to a more realistic case i.e., with a model trained on past data and evaluated on the current ones.

Note that although validation on the latest data (i.e., in col. 6), while being closer to a real usage scenario, still has one systemic drawback. Groups of URLs in the dataset are made based on all available requests in history, while in reality model is to work on completely unseen data, potentially not matching any of the previously made groups. If the importance of this observation were to be assessed, another subset, a test subset made of the very latest data should have been carved out and used. We considered such an overarching evaluation of the scope of our study, first, because our data has a strong trend indeed, which we believe is not so prominent in, e.g., an online banking case. Second, because the data used by us is rather scarce—however, in Section 3.2, we address the issue and propose ways to extend the usable dataset.

Table 2 shows the results of the models split according to the input data format and the network architecture used. We provide the input data either without time (basic) or with time (extended). An example of data without time is simply a sequence of five queries generated by the user at a certain time (as mentioned earlier), stored as a sparse, one-hot-encoded matrix. In the case of data with time, each of the input matrices representing a given session was expanded by adding a row with logarithms of inter-request times.

As for the breakdown by network architecture, classical Recurrent Neural Networks (RNN) and their extension, i.e., Gated Recurrent Units (GRU) and LSTM, were considered as the second layer of the model in the course of experiments. Recurrent neural networks are well-suited to handle sequential data and meet our goals. However, they suffer from a vanishing gradient problem, which requires special extensions to address it. GRU and LSTM use special memory cells to store information and gates to control which transitions in the sequence are important enough to store.

Evaluation results in Table 2 for manual URL grouping are not satisfactory. None of the configuration options provide accuracy that is significantly better than its counterpart, and the accuracy reaches at most 65% for the LSTM variant with dynamics included, which is definitely too low for a standalone solution. However, the model can already provide useful scores as a part of a more complex intruder detection system. From a technical point of view, it is also worth observing that validation done in the 6th period yields results lower than average for LSTM and GRU variants—but the differences are minor.

### 4.2. Models Trained on Data After Automated Grouping of URLs

Results in Table 3 show that automated grouping of URLs leads to models of consistently much higher quality of at least 10 percentage points over any result in Table 2, and of at least 17 percentage points in mean terms. All configurations, except RNN with user dynamics, are of equal quality, with differences safely within the standard deviation. The similarity of the results across configuration parameters may indicate that proper dataset quality and preprocessing are key factors in model effectiveness.

### 4.3. Model Verification with User Galleries and Other Insights

Although classification models like ours are designed to compare a current session with a reference one, in industry practice they are employed to compare the current session with each of a user’s historical sessions, stored in a so-called gallery—cf. [7,16]. In Table 4, we provide model accuracy computed in such a way. Each validation sample $x_{i}\in V$ was compared with all training samples by user *c* that constitute the gallery, i.e., $x_{j}^{\prime }\in T_{c}$, producing similarity scores $d_{ij}$. Sample $x_{i}\in V$ was classified as genuine only if the mean of scores was above threshold μ, and the maximum of scores was above threshold θ. The parameters were set to μ=0.6 and θ=0.5, which is rather a strict setting with regard to μ, which is the condition that is often dropped in industry applications.

Obtained results systematically beat previous ones and consistently point to the GRU model with user dynamics included as the best one. The mean accuracy score nears 90%, which we consider a border value for standalone practical applications.

We also decided to perform a minimal ablation study by starting with the best model configuration and removing the dropout layer from our architecture. The results are summarized in Table 4, where the last row, in italics, represents the dropout ablation within ‘GRU+time’ variant. We observe a minimal yet almost consistent (save for Period 3) performance decrease, which proves that the application of best engineering practices adds value to the model—especially that the dropout layer does not burden the training with extra parameters.

Other experiments, shown in italics in Table 4, examine the sensitivity of the best model with regard to the interarrival cutoff time (initially, of 256 s), and to the increased number of users (initially, 10). Shortening the packet inter-arrival time that determines session continuity from the initial 256 by 10 percent yielded results shown in row ‘−10% cutoff time’. Except for Period 3 again, the accuracy decreases by a minimal amount. Due to the highly discrete impact of the cutoff value on raw data filtering, one should expect high variability in results when this parameter is reduced more aggressively.

By relaxing the minimum number of login sessions per user from 24 to 18, we were able to increase the number of valid users from 10 to 15 and assess how this affected the accuracy of the best model. The results are provided in row ‘+50% users’. The accuracy falls almost consistently across folds, with a mean change of 3.6 percentage points. Alternatively, if we take a complementary approach, the ratio of misclassified samples increases by (100−83.9)/(100−87.5)≈1.29, with the number of users growing by a factor of 1.5. While the accuracy deterioration with a growing user population is undeniable, its character is not proportional to the population, and, with the data available, we cannot examine it at scale. It should also be noted that, due to data scarcity, the number of sessions per user in the larger population had to be limited, contributing negatively to the results.

**Figure 5:**
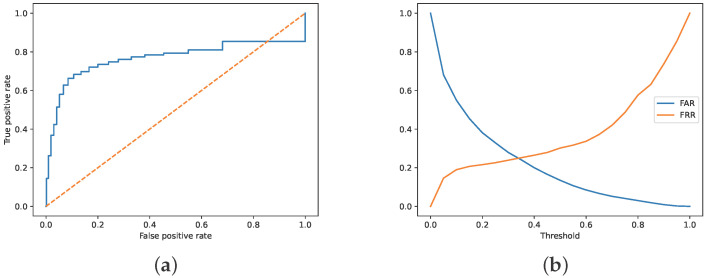
(**a**) ROC curve (blue, vs. dashed random-guess line); (**b**) false acceptance rate (FAR) and false rejection rate (FRR) plotted versus the similarity threshold. The equal error rate (EER) is shown as a cross point of FAR and FRR.

Ultimately, the interpretation of classifier effectiveness depends on its usage context. For non-critical applications, such as mail spam filters, specificity will be the goal—lest false-positive messages be lost, even at the cost of much spam passing through the filter. In critical applications, like luggage security scans, sensitivity will be the goal because security staff can easily examine false positives. The trade-off between sensitivity and specificity is typically drawn as a receiver operating characteristic (ROC) and false acceptance vs false rejection rate graphs (FAR vs FRR). Such graphs are provided for the best-performing model evaluated on the latest user activity (accuracy of 78.1% in Table 3) in Figure 5. As explained in Section 4.1, such a method of evaluation resembles real operation the most. Both graphs look typical, with area under curve (AUC) value of 0.8 for ROC. The equal error rate (EER) for FAR and FRR is around 0.35. However, the best accuracy is observed for a threshold value of 0.5—i.e., the default value for which comprehensive model evaluation in Table 2 and Table 3 was performed.

It is worth noting that the FAR graph is more slanted for ERR than for FRR, which means that raising the threshold to 0.5 may substantially improve specificity at the marginal cost of reduced sensitivity. While the increased sensitivity may be annoying in the spam-filtering scenario mentioned above, it is indeed quite acceptable in the banking scenario, where extra user authentication is now commonplace and compliant with policies requiring out-of-band verification for transactions [31].

**Figure 6:**
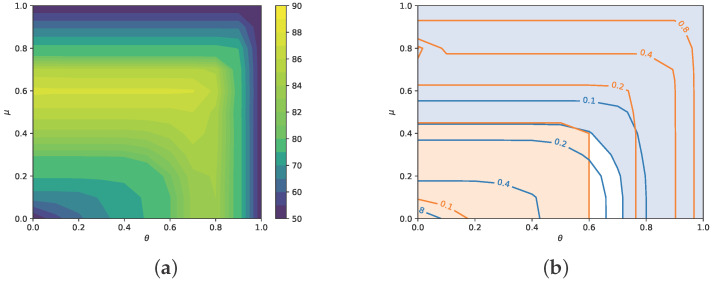
Model evaluation results for operation on user galleries, with threshold parameters μ for the mean similarity, and θ for best similarity to probes in the gallery. (**a**) Model accuracy (in percent). (**b**) FAR (blue) and FRR (orange) contour plots.

In the case of model evaluation against user galleries, as summarized in Table 4, we introduce actually two threshold types, μ and θ. Consequently, model accuracy and FRR/FAR analyses become two-dimensional, as shown in Figure 6. The layout of both graphs helps to assess at a glance the importance of both thresholds. As for the accuracy (Figure 6a), we observe the dominant role of the mean similarity to gallery probes, providing the best results for μ=0.6, with no real impact of θ (until θ≥0.7 where the accuracy degrades). The result is contrary to application practice, which typically seeks the maximum similarity to any gallery item.

The analysis is further split into FAR/FRR graphs in Figure 6b, being a 2D counterpart of Figure 5b. In the two-dimensional case, there can be many (μ,θ) pairs resulting in ERR. An important part of such (μ,θ) set displayed in the graph is the common border of the two shaded areas—the orange and gray ones denoting small FRR and FAR, respectively. The border, lying at μ≈0.48, marks an ERR of 0.155. Going further to the right, the two shaded areas diverge, meaning, in our case, that ERR is increasing and stays in the uncolored, hence less favorable, area. A two-dimensional EER imaging like this one may help decide about useful threshold values in an actual application scenario, analogously to Figure 5b.

## 5. Conclusions

The aim of the research reported here was to construct a behavioral model that classifies pairs of HTTP sessions as originating from the same user or two different users. Our work was inspired by similar studies made on various behavioral modalities (keyboard, mouse, URL request—cf. Section 2) as well as the fact that the model class of Siamese networks has also been successfully used in similar tasks focused on URL semantics. Our contributions are as follows:

- collecting and post-processing of standard WWW server logs from a real environment;
- development and testing of the Siamese network model;
- proposal and testing of automated grouping of URLs, based on low-rank matrix approximation;
- releasing of the dataset and code related to model training on automatically grouped URLs to the public.

The novelty of the proposed algorithm lies in the automated clustering of URLs in abstract space inspired by low-rank factorization used in recommender systems. The approach relieves web service operators of the manual investigation of weblogs to perform grouping tailored to the current website configuration. The algorithm’s similarity score can be (and should be) fused with other modality-specific models to improve overall quality. Additionally, the Siamese network model does not encode traits of individual users in any way and preserves user privacy most, to the best of our knowledge.

The data pre-processing phase revealed that special account must be taken for machine-to-machine communication, thereby obliterating the user’s spontaneous activity. The particular extra traffic pattern will heavily depend on the WWW framework running on the server; one has to be wary that in the extreme case, no or very little traffic can be observed on standard HTTP/HTTPS ports as the bulk of communication is going via dedicated channels that are unknown to WWW server. This, however, can be accounted for by investigating auxiliary logs, depending on the framework used.

The procedure for the automated grouping of requested webpages derives directly from data compression and user preference generalization, first applied in text information retrieval and later in recommendation systems. The low-rank approximation is now an established unsupervised machine learning approach that is fast and effective in many fields. Our data pre-processed this way has proven to be of better quality for the chosen model class, outperforming the standard manual approach by at least 10 percentage points in terms of model accuracy. All three examined model structures handle sequences in the input layer and perform similarly. Providing inter-request dynamics as additional model input data improves performance only marginally.

One has to take into account that remarkably good accuracy was obtained in this study only for a moderate number of users, allowing them to be distinguished with only five requests per session. We are aware that, if deployed in a large organization, the model would perform worse and have no potential to improve due to tight limits on session length. Fraud detection on longer sequences would be more effective, but fraud prevention would be less efficient, as longer sequences leave an intruder more space to complete a fraud. Still, our approach would be attractive, possibly as a part of a bigger IDS, and for other security-related reasons. This is because individual user behavior traits are not embedded into the Siamese network; rather, the model learns which parts of session information best distinguish users. Consequently, the only real user-related information that needs to be collected to identify him/her later is the history of past sessions. A service operator processes such short sequences of requests in the gallery solely to accomplish user identity verification. Therefore, the proposed approach performs processing user activity data in a minimal way and does not perform personal data profiling at all (following definitions in Articles 4 and 25 of General Data Protection Regulation, GDPR.eu). The strive to conform to GDPR regulations, which require an operator to store only data relevant to its tasks, is evident in medical studies. Interestingly, depending on the training approach, Siamese networks can be used there malevolently to identify patients’ identity from medical imagery, as reported by [32], as well as to rightfully detect specific patients’ traits (e.g., sex) yet obliterating other features so that patient identity stays protected—cf. [33].

Observations of this kind lead us to another, final conclusion: model robustness against a kind of adversarial reverse engineering aimed at extracting bits of the training data should be measured and considered another important model parameter, along with sensitivity, specificity, and the like. While defining measures and evaluation of robustness of our model in quantitative terms is beyond the scope of this study, we can assess weaknesses of our approach with regard to the typical attack methods. First and foremost, since the Siamese model operates on the backend, access to its weights, vector outputs, user gallery, or intermediate vector distances is impossible. Therefore, typical attack approaches to an existing model, such as weight stealing, approximating the output vector space, or constructing a reverse mapping from the input space to the output space, remain out of question. Next, poisoning the model during training with manipulated samples could be considered effective only if carried out on a massive scale and in a coordinated manner. In such a scenario, an attacker would behave differently in each session, adding noise to authentic behavioral patterns and impairing the model’s sensitivity. It can be addressed by identifying outlier users and excluding their inputs from model training. Finally, monitoring network traffic appears to be the ultimate attack strategy: the attacker observes the visited addresses and their timing to impersonate the victim’s behavioral traits. However, while the request timing is indeed observable, only the URL domain name is visible during an HTTPS session. Therefore, the only way to inspect the details of request timings is to examine either the client’s browser history or the web server’s log.

While the empirical results show that the proposed design approach leads to a solution that appears stable under small perturbations in the operating environment (e.g., the number of users or the session inactivity limit), the analogy between online banking and intranet activity data is approximate. Phenomena such as the trend and architectural changes may differ in the real banking scenario from those in this study, requiring additional data preprocessing or more frequent model training.

The ongoing work following this study is focused on practical verification of the model presented in the online banking scenario. Also, more complex strategies of comparing the current session with a set of historical ones are under investigation. Early results from other application domains show that learning the comparison strategy is a promising future research direction.

## Figures and Tables

**Table 1:** Exemplary functional and technical analogies between a corporate website and three national banks, ordered in rows by decreasing similarity. Functional analogies are written in regular font. Technical similarity is conveyed through italicized references to specific implementation and communication techniques. The last two rows demonstrate exemplary actions that have no counterparts.

User Action on the Intranet Website	User Action on Website of		
	Bank#1	Bank#2	Bank#3
log in	log in		
*a*	*a*	*aeg*	*a*
look up a person	look up a beneficiary		
*a*	*f*	*ac*	*df*
free-text search	search past money orders		
*bf*	*bf*	*ac*	*df*
submit an opinion	ask for help		
*ac*	*abdg*	*ad*	*abdg*
mention someone in a post	place money order to someone		
*bd*	*abc*	*ac*	*cf*
—	buy/sell currency		
	*acg*	*cef*	*ceg*
display organization scheme	—		
*g*			

*a*—form data is sent to the server only after the “send” button has been pressed; *b*—form data is sent during typing; *c*—multifield static form is used; *d*—a form with a single text field is used; *e*—partial communication happens with more than one servers; *f*—form data is fully or partially pre-loaded; *g*—form or page content changes dynamically, in response to user actions.

**Table 2:** Comparison of accuracy, in percent, of models with various types of the recurrent 2nd layer (RNN/LSTM/GRU) and in variants when browsing dynamics are, or are not, included (time/no time). All models were trained on data with web requests grouped manually. Numbered columns contain results for validation subsets $V_{1}$ to $V_{6}$, respectively (cf. Figure 2a). The best solution is marked in bold face.

Model Variant		Period Used for Validation						Mean	Stdev
		1	2	3	4	5	6		
RNN	no time	52	49	51.6	56	54.7	53.8	52.9	4.9
	time	49.3	51	56.6	56.4	54.4	54.8	53.8	5.2
LSTM	no time	63.7	64.4	64.8	59.7	60.9	56.3	61.6	4.1
	time	64.7	**65.3**	64.8	60.3	62.4	57.2	62.4	3.8
GRU	no time	59.9	62.6	62	62.1	61.7	57.5	61	4.9
	time	61.6	60.9	65	61.8	63.1	58.1	61.7	4.9

**Table 3:** Accuracy of models trained on data with web requests grouped automatically. Key best solutions are marked in bold face.

Model Variant		Period Used for Validation						Mean	Stdev
		1	2	3	4	5	6		
RNN	no time	83.6	81	83.4	74.4	76.7	75.4	79.1	4.5
	time	74.3	70.3	76.9	73.8	70.6	70.0	72.6	4.1
LSTM	no time	80.6	79.9	84.2	75.3	79.8	74.2	79.0	4.6
	time	81.0	80.3	**85.1**	76.0	80.4	74.7	79.6	4.4
GRU	no time	83.6	81.1	83.5	74.4	76.6	75.5	79.1	4.5
	time	83.1	80.5	82.7	76.7	77.3	**78.1**	**79.7**	3.6

**Table 4:** Accuracy of models trained on data grouped automatically, calculated by comparison on validation samples against a user gallery consisting of training samples. Sensitivity of the optimal model with regard to its structure and data pre-processing rules, is provided in the last three rows.

Model Variant		Period Used for Validation						Mean	Stdev
		1	2	3	4	5	6		
RNN	no time	91.4	88.5	88.1	80.2	86.2	84.8	86.5	4.7
	time	83.4	78.5	84.3	79.3	77.8	83.4	81.1	4.8
LSTM	no time	88.2	86.1	88.8	81.4	87.3	83.9	85.9	4.4
	time	86.7	86.6	89.3	82.2	87.4	85.2	86.2	3.6
GRU	no time	91.4	88.3	88.1	80.2	86.2	85	86.5	4.7
	time	90.7	89.3	88.5	82.8	86.8	**87.1**	**87.5**	3.9
	*−10% cutoff time*	*88.8*	*89.2*	*89.6*	*83.3*	*86.3*	*84.5*	*86.9*	*3.1*
	*+50% users*	*81.8*	*83.6*	*81.9*	*89.6*	*86.7*	*79.8*	*83.9*	*4.0*
	*no Dropout*	*88.6*	*89.1*	*90.1*	*81.7*	*86.5*	*87.0*	*87.2*	*3.9*

## Data Availability

The dataset of web user activity and source code excerpts are available at DOI:10.5281/zenodo.10670928.

## References

[1] Solo A.M. Combating Online Impersonation in the United States. Proceedings of the 2019 International Conference on Computational Science and Computational Intelligence (CSCI).

[2] da Silva R. (2021). Calls for behavioural biometrics as bank fraud soars. Biom. Technol. Today.

[3] U.S. Federal Trade Commission (2023). Report on the FTC’s Top Management and Performance Challenges. https://www.ftc.gov/system/files/ftc_gov/pdf/OIG-FY-2023-Top-Management-Challenges-Report-09-29-2023.pdf.

[4] Karim N.A., Khashan O.A., Kanaker H., Abdulraheem W.K., Alshinwan M., Al-Banna A.K. (2024). Online Banking User Authentication Methods: A Systematic Literature Review. IEEE Access.

[5] Kałużny P. (2019). Behavioral Biometrics in Mobile Banking and Payment Applications. Proceedings of the Business Information Systems Workshops.

[6] Bromley J., Guyon I., LeCun Y., Säckinger E., Shah R. (1993). Signature verification using a “siamese” time delay neural network. Adv. Neural Inf. Process. Syst..

[7] Dahia G., Jesus L., Pamplona Segundo M. (2020). Continuous authentication using biometrics: An advanced review. Wiley Interdiscip. Rev. Data Min. Knowl. Discov..

[8] Mahadi N.A., Mohamed M.A., Mohamad A.I., Makhtar M., Kadir M.F.A., Mamat M. (2018). A survey of machine learning techniques for behavioral-based biometric user authentication. Recent Advances in Cryptography and Network Security.

[9] Ryu R., Yeom S., Kim S.H., Herbert D. (2021). Continuous Multimodal Biometric Authentication Schemes: A Systematic Review. IEEE Access.

[10] Shende S.W., Tembhurne J.V., Ansari N.A. (2024). Deep learning based authentication schemes for smart devices in different modalities: Progress, challenges, performance, datasets and future directions. Multimed. Tools Appl..

[11] Yi Q., Xiong S., Wang B., Yi S. (2020). Identification of trusted interactive behavior based on mouse behavior considering web User’s emotions. Int. J. Ind. Ergon..

[12] Wang B., Zhai Z., Gao B., Zhang L. (2021). Identity Recognition Based on the Hierarchical Behavior Characteristics of Network Users. Proceedings of the International Conference on Human-Computer Interaction.

[13] Giot R., Rocha A. Siamese networks for static keystroke dynamics authentication. Proceedings of the 2019 IEEE International Workshop on Information Forensics and Security (WIFS).

[14] Lis K., Niewiadomska-Szynkiewicz E., Dziewulska K. (2023). Siamese Neural Network for Keystroke Dynamics-Based Authentication on Partial Passwords. Sensors.

[15] Stragapede G., Vera-Rodriguez R., Tolosana R., Morales A., DeAndres-Tame I., Damer N., Fierrez J., Garcia J.O., Gonzalez N., Shadrikov A. IEEE BigData 2023 Keystroke Verification Challenge (KVC). Proceedings of the 2023 IEEE International Conference on Big Data (BigData).

[16] Ayotte B., Banavar M., Hou D., Schuckers S. (2020). Fast Free-Text Authentication via Instance-Based Keystroke Dynamics. IEEE Trans. Biom. Behav. Identity Sci..

[17] Varnosfaderani S.D., Kasprzak P., Pohl C., Yahyapour R. SmartSSO—A Deep Learning Platform for Automated Account Linkage in Federated Identity Management. Proceedings of the 2021 IEEE International Conference on Omni-Layer Intelligent Systems (COINS).

[18] Milton L., Robbins B., Memon A. N-gram-based user behavioral model for continuous user authentication. Proceedings of the SECURWARE 2014.

[19] Li J., Yi Q., Yi S., Zhu P. (2022). Developing Virtual Fingerprint: Identifying Trustworthy Interaction Behaviors of Web Users. https://www.researchsquare.com/article/rs-1792004/v1.

[20] Jin D., Heimann M., Rossi R.A., Koutra D. (2019). node2bits: Compact time-and attribute-aware node representations for user stitching. Proceedings of the Joint European Conference on Machine Learning and Knowledge Discovery in Databases.

[21] Charikar M.S. Similarity estimation techniques from rounding algorithms. Proceedings of the Thiry-Fourth Annual ACM Symposium on Theory of Computing.

[22] Sudhakar K., Smail B., Reddy T.S., Shitharth S., Tripathi D.R., Fahlevi M. Web User Profile Generation and Discovery Analysis using LSTM Architecture. Proceedings of the 2022 2nd International Conference on Technological Advancements in Computational Sciences (ICTACS).

[23] Qiao Y., Wu Y., Duo F., Lin W., Yang J. (2019). Siamese neural networks for user identity linkage through web browsing. IEEE Trans. Neural Netw. Learn. Syst..

[24] De Smedt J., Lacka E., Nita S., Kohls H.H., Paton R. (2021). Session stitching using sequence fingerprinting for web page visits. Decis. Support Syst..

[25] Panda C. (2020). Permanent Revolution: How Micro Front Ends Can Help Banks Overcome the Challenge of Continuous Modernization. https://tinyurl.com/yxdyeyt3.

[26] Lang K., Hinton G. (1988). A Time-Delay Neural Network Architecture for Speech Recognition. The echnical Report CMU-cs-88-152. Ph.D. Thesis.

[27] Sharma V., Tapaswi M., Sarfraz M.S., Stiefelhagen R. Self-supervised learning of face representations for video face clustering. Proceedings of the 2019 14th IEEE International Conference on Automatic Face & Gesture Recognition (FG 2019).

[28] Gomez J.A.S., Ronderos M.O., Guerrero E.R., Cruz A.C., Hurtado L.T., Escobar C.D.L. (2024). One-Shot Behavioral Biometrics for Login Authentication Using Machine Learning Model.

[29] Wahab A., Hou D. Impact of Data Breadth and Depth on Performance of Siamese Neural Network Model: Experiments with Two Behavioral Biometric Datasets. Proceedings of the 22nd International Conference of the Biometrics Special Interest Group.

[30] Koren Y., Bell R., Volinsky C. (2009). Matrix factorization techniques for recommender systems. Computer.

[31] Council, Federal Financial Institutions Examination (2011). Supplement to Authentication in an Internet Banking Environment. https://www.occ.gov/news-issuances/bulletins/2011/bulletin-2011-26a.pdf.

[32] Esmeral L., Uhl A. (2022). Low-effort re-identification techniques based on medical imagery threaten patient privacy. Proceedings of the Annual Conference on Medical Image Understanding and Analysis.

[33] Nelus A., Rech S., Koppelmann T., Biermann H., Martin R. Privacy-Preserving Siamese Feature Extraction for Gender Recognition versus Speaker Identification. Proceedings of the INTERSPEECH 2019.

